# Development of Natural Fibre-Reinforced Semi-Finished Products with Bio-Based Matrix for Eco-Friendly Composites

**DOI:** 10.3390/polym14040698

**Published:** 2022-02-11

**Authors:** Claudia Möhl, Timo Weimer, Metin Caliskan, Stephan Baz, Hans-Jürgen Bauder, Götz T. Gresser

**Affiliations:** 1 German Institutes of Textile and Fiber Research (DITF), Körschtalstrasse 26, 73770 Denkendorf, Germany; claudia.moehl@ditf.de (C.M.); timo.weimer@ditf.de (T.W.); metin.caliskan@ditf.de (M.C.); stephan.baz@ditf.de (S.B.); hans-juergen.bauder@ditf.de (H.-J.B.); 2 Institute for Textile and Fiber Technologies (ITFT), University of Stuttgart, Pfaffenwaldring 9, 70569 Stuttgart, Germany

**Keywords:** natural fibre, bio-based thermoplastics, hybrid yarn, woven fabric, fibre waste, textile waste, mechanical characterisation

## Abstract

Increasing resource consumption and a growing amount of textile waste increase the importance of a circular economy and recycling in the fashion and apparel industry. Environmentally friendly bio-based composites made from cellulosic fibres obtained from textile waste, and polymers based on renewable raw materials present a possible solution. In this study, the development of textile semi-finished products based on medium-to-long cotton and flax fibres obtained from textile waste in combination with a bio-based thermoplastic matrix for lightweight applications is investigated. For the production of natural fibre-polylactide hybrid yarns, fibre slivers with improved fibre orientation and blending are produced. Subsequently, quasi-unidirectional woven fabrics are produced and consolidated into bio-based composites. Textile and mechanical properties of hybrid yarns as well as bio-composites are analysed with regard to the influence of fibre length, fibre distribution in the yarn, yarn structure and fibre volume content. The results show that the production of bio-based semi-finished products can be a potential way for upcycling textile waste.

## 1. Introduction

As a result of growing environmental awareness, concerns about environmental sustainability and the growing global waste problem are becoming more pressing every year. The steady and continuous growth of the textile market drives the textile industry to produce more and more textiles every year to meet this demand [1]. Besides the growth of the population, the shortening of fashion cycles and the low price of textiles compared to other consumer goods are major reasons for this trend [2]. Correspondingly, consumer behaviour in the European Union has changed to such an extent that the purchase rate for clothing and textiles has increased by 40% in less than twenty years, according to Dahlbo et al. [3]. The challenges of dealing with environmental, energy and resource issues are increasing as the textile industry grows. A large proportion of textiles (around 63%) are made of synthetic fibres, which are mainly produced from petrochemicals and cause significant CO_2_ emissions during their production [3,4,5]. This is followed by cotton, the production of which requires a significant amount of water and often involves the use of pesticides [6]. Globally, the largest share of textile waste is not recycled, but either incinerated or used in landfills. This causes additional environmental problems such as long disposal times, the emission of pollutants into surrounding soils during decomposition, the release of carbon dioxide as well as methane and the potential contamination of groundwater. Only a very small proportion of recyclable textiles are reused through downcycling routes, which results in products with lower quality and thus limiting further applications in technical fields [4,5,7]. In this way, huge quantities of cellulosic fibres are wasted every year and end up in landfills, even though their attractive properties could make their use in geotextiles and composites worthwhile.

Since, in a circular economy, great importance is attached to maintaining or even increasing the value of the recycled products, various attempts have been made at the academic and industrial level to incorporate different cellulosic fibres from textile waste into thermoplastics [8,9,10], thermosets [11,12,13] or concrete [14,15,16] to enable specific applications such as sound-absorbing, partial load-bearing building elements, filter membranes, furniture and automotive components.

In terms of so-called “green” composites, in addition to environmentally friendly bio-composites made from cellulosic fibres and thermoplastic polymers based on renew-able raw materials, studies are also being conducted on the use of textile waste. Due to the special properties of cotton fabrics such as large, deformable surface, low weight and good flexibility, Chen et al. combined the textile waste with natural rubber to develop flexible and skin-friendly strain sensors for measuring muscle contractions [17]. To develop a sound-absorbing material, Dissanayake et al. used a composite of waste cotton knit, waste polyester yarn and natural rubber [18]. Their results show that the sound insulation properties are comparable to those of commercial sound insulation panels, and the sound insulation properties increased with increasing panel thickness. In the study by Montava-Jorda et al., fibres made from recycled cotton are first shredded and then melt-compounded with partially bio-based polyethylene terephthalate (Bio-PET) using a twin-screw extruder [19]. In the range of 1–10 wt.% cotton waste, the stiffness and rigidity of the Bio-PET is successfully increased. However, ductility and toughness are significantly reduced, especially at the highest fibre mass content, due to the poor interfacial adhesion of the fibres to the bio-polyester matrix. The authors also observed that the cotton waste acts as an effective nucleating agent for the Bio-PET crystallisation, thus increasing the thermal resistance. Furthermore, the overall dimensional stability of the samples is improved, depending on the fibre content. Bio-PET samples with 3 to 5 wt.% cotton waste exhibited very balanced properties in terms of mechanical strength, toughness and thermal resistance. The resulting bio-polymer composites may therefore be of interest for rigid food packaging and related applications. Bajracharya et al. analysed the mechanical properties of polylactide filled with cotton bur stem (cotton gin waste) and flax fibre particles [20]. The addition of 30 wt.% cotton bur stem increased the flexural modulus by 42% compared to pure polylactic acid (PLA). Furthermore, the elastic modulus of the cotton bur stem composites improved comparably to the hybrid composites additionally filled with different percentages of flax fibre particles. The work of Araújo et al. deals with the hydrolysis of cotton fibres from textile waste to obtain micro- and nano-fibres that can be used as reinforcement in polymer composites, and the improvement of fibre-matrix adhesion to polylactide by modifying the surface using different silane compounds [21]. The cellulose fibres hydrolysed in two steps significantly improved the thermal stability of the compound. However, fibre diameter and length decreased due to the hydrolysis process. The increase in the modulus of elasticity and the breaking stress of the composite compared to the pure matrix is therefore mainly due to silanisation rather than an improved interfacial adhesion between polymer and fibre. In the overall results, the authors conclude that cotton waste can be effectively recycled as fillers in polymer-based composites, through appropriate surface modification strategies.

In the aforementioned studies, the textile waste is presented in the form of woven fabrics or very short fibres down to particle and nano size. The aim of this work is to develop an environmentally friendly and cost-effective semi-finished product for fibre-reinforced bio-based composites based on medium-to-long cotton and flax fibres from textile waste, which are obtained in a recycling process. The process for the production of the hybrid yarns is described in detail in the following sections. The aim is to mix and combine the natural fibres and thermoplastic components as homogeneously as possible, so that the flow distance of the matrix during consolidation can be reduced and better mechanical properties of the composites can be achieved. Afterwards, the hybrid yarns are further processed into quasi-unidirectional (quasi-UD) woven fabrics. Special focus is placed on the alignment and to ensure the least possible undulation of the yarns, in order to achieve the best possible mechanical characteristics for the composites. The quasi-UD woven fabrics are consolidated into composite plates in a hot pressing process and then characterised in terms of their tensile and bending properties.

## 2. Materials and Methods

For the manufacturing of the bio-based composites, textile semi-finished products are first produced in the form of hybrid yarns and processed further into woven fabrics. The woven fabrics are then cut, stacked and consolidated into bio-based composite materials by using hot pressing process. Tensile and four-point bending tests are carried out to analyse the mechanical properties of the corresponding samples. In the following section, the most important points regarding the spinning and weaving technology used, as well as the composite manufacturing process, are explained.

### 2.1. Material

In this study, cotton fibres (CO) and flax fibres (FL) from different stages of spinning processes, as well as recycled from end-of-life-textile waste, in combination with polylactic acid (PLA) IngeoTM fibres (38 mm length) and PLA IngeoTM filament yarn from TREVIRA^®^, Bobbingen, Germany, are used for the production of hybrid yarn and woven fabrics. The main properties of these materials are shown in Table 1.

### 2.2. Spinning Process and Parameters

#### 2.2.1. Carding and Drawing

For the manufacturing of slivers consisting of either waste cotton fibres or waste flax fibres and PLA fibres, both components are pre-opened and blended in an opener in a ratio of 50 wt.% each (10 kg in total). To form a card sliver of 5.5 ktex, the fibres are fed from the bale opener type CS over a dust extractor type DustEx into a card type Dk715. All three machines are from Trützschler GmbH & Co. KG, Mönchengladbach, Germany. In order to produce slivers with higher regularity, high oriented and improved mixing of fibres six card slivers were fed in parallel to a draw frame RSB-D35 from Rieter Holding AG, Winterthur, Switzerland. To compensate for the variations in individual slivers even further, six slivers of the first draw frame were fed in parallel on another draw frame (type RSB-D45 from Rieter Holding AG, Winterthur, Switzerland) with active sliver weight control and processed into a draw frame sliver of 5 ktex.

#### 2.2.2. Spinning of Hybrid Yarns

**Figure 1:**
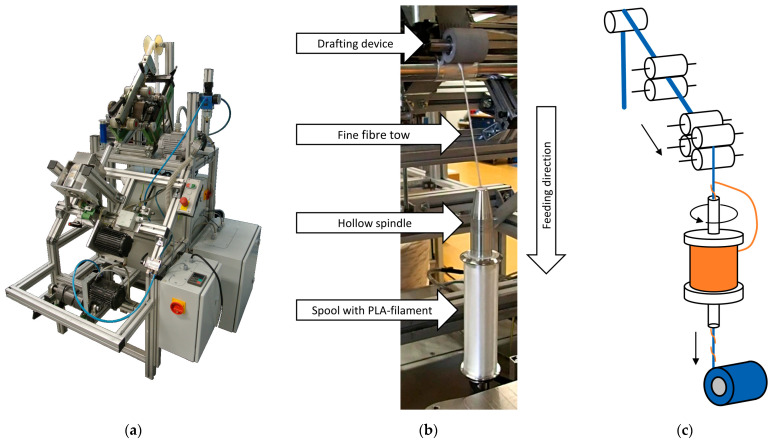
(**a**) Self-built filament-wrap spinning machine; (**b**) Details of filament wrapping process; (**c**) Schematic of wrap spinning process.

Hybrid yarns with a fineness of 800 tex are spun using filament wrap spinning on a self-built machine (Figure 1a). In this process, the draw frame slivers are fed to a drafting device and drawn to form a finer fibre tow. Due to the drafting of the fibre slivers starting from 5 ktex to the final fineness of 800 tex, a high degree of fibre orientation is achieved. The fibre orientation process is supported by the narrowing of the cross-section occupied by the fibre mass, which increases the draft and therefore the fibre orientation. The output of the drafting device is then wrapped with a filament (Figure 1b). The spool with wrapping filament is mounted on a hollow spindle. The winding of the core takes place as the hollow spindle and, thus, the spool with the filament, rotates, while at the same time the sliver is drawn off the drafting system. Each revolution of the wrapping filament around the sliver corresponds to one complete revolution of the filament spool (Figure 1c). The spinning process was selected because the resulting yarn core with the natural fibres is almost torsion-free and thus the potential of the reinforcement with high oriented fibres can be optimally utilised. In addition, the wrapping filament rises the pressure on the fibres of the core and thus increases the fibre–fibre friction to sustain the tension loads applied during the weaving process [22].

The drafting system setup and settings were chosen based on experience from previous studies and on the basis of measured fibre length. The drafting system consists of a three-cylinder setup with double aprons in the main draft resulting in two draw fields (break and main draft). The field widths were chosen according to the measured value of the fibre length measurement, which describes which length 5% of the longest fibres have. For cotton, this was 38 mm, and for flax, 45 mm. A field width of 40 mm was selected as the starting value. In order to obtain a comparability of the drafting system setting in the composite, both fibre types were processed with the same drafting system settings. However, initial tests showed irregular, slightly wavy drafting of the fine fibre tow and consequently increased sliver breaks between the drafting system and the entry into the hollow spindle. This was caused by the large variation in lengths between the shortest and the longest natural fibre. By changing the drafting system to process fibre length from 40 mm to 50 mm, the drafting behaviour of the draw frame was significantly improved.

The subsequent step was to determine suitable settings for the main drafting system draft and the number of turns per meter. While the main draft determines the fineness of the resulting yarn, the wraps influence the volume as well as the yarn strength. The fewer the number of wraps around the core, the more voluminous and less strong the yarn. The best results in terms of evenness and fineness of the hybrid yarn were achieved with a main draft of 4.2 fold and a wrap count of 150 turns per meter.

The most important parameters of the spinning process and the manufactured hybrid yarns are represented in Table 2.

### 2.3. Weaving Process and Parameters

The classification “eco-friendly” is becoming more and more popular with all kinds of products. Ecological friendly fibres reinforced composites can be manufactured using natural fibres as reinforcement and natural polymers as matrix material [23]. The fibres used in composite materials are mostly available as fabrics. The fabrics are generally knitted, braided, stitched or woven. Weaving is the most used process to produce fabrics for composites because of its high productivity and flexibility [24]. Furthermore, weaving can produce transverse and longitudinal reinforced (biaxial) semi-finished goods, for fibres products. The weaving technology makes it possible to bring the fibres in a textile surface from a very dense structure to an open grid structure [25].

One of the main characteristics of semi-finished materials is their mechanical behaviour during manufacturing processes. Due to the integration of thermoplastics, hybrid yarns behave differently when woven. The weaving process needs to be tailored to the specific characteristics of the yarn.

**Figure 2:**
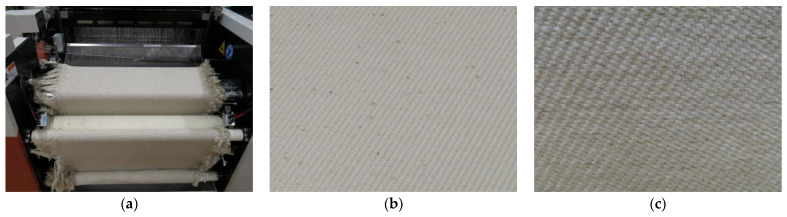
(**a**) Production of the quasi-UD fabric, insertion of the weft yarn; (**b**) quasi-UD CO-PLA fabric; (**c**) quasi-UD FL-PLA fabric.

The weaving process took place on the sample weaving loom, Evergreen II from CCI Tech Inc., New Taipei City, Taiwan. The weaving loom has a rapier insertion system for the weft and a positive let off system for the warp beam (Figure 2a). The warp beam is made on the mini warping machine SW 550, also from CCI Tech Inc., New Taipei City, Taiwan. The speed and the yarn tension during the direct warping process are to be adjusted to avoid yarn breakages and to ensure a homogeneous stress distribution throughout the warp beam. The maximum length of the warp yarns is limited to 3.60 m due to the mechanical limitations of the mini warping machine. The maximum possible length of the finished fabric is around 2.40 m, considering the machine related losses. The losses come from the adjusting of the machine and the distance between the warp beam and the weaving reed.

During the weaving process, Ashir et al. recommend the exact adjustment of the raising and lowering of warp yarns, in order to compensate different yarn undulations and, therefore, differences in length [26]. Both fabrics are woven with the identical parameters given in Table 3. The results are two quasi-unidirectional woven fabrics with twill 2/2 binding. The first produce fabric is the quasi-UD cotton-PLA fabric (Figure 2b), the second is the quasi-UD flax PLA fabric (Figure 2c). Quasi-unidirectional fabrics have a very high fibre orientation in one direction. The unidirectional fibres are fixed in their position by fibres in one other direction and are nearly uncrimped. Therefore, quasi-unidirectional woven fabrics come very close to the case of stretched, unidirectional fibre alignment and, thus, meet the requirements for structural applications [27].

### 2.4. Composite Frabrication

Bio-based composite plates based on waste cotton-PLA woven fabric and waste flax-PLA woven fabric were manufactured using a vacuum hot press VCP500 from Maschinenfabrik Lauffer GmbH & Co. KG, Horb, Germany. All quasi-UD woven fabrics were cut to 400 mm × 400 mm plies and dried in oven at 80 °C for at least 2 h. Four plies each were stacked to form a unidirectional composite plate (0°). Then, they were placed between two Teflon-coated plates and positioned in the hot press.

In order to determine suitable pressing parameters for the unidirectional woven fabrics, preliminary tests were carried out on quasi-UD waste cotton fibre-PLA and quasi-UD waste flax fibre-PLA woven fabrics at the beginning of the experiments. For this purpose, test pieces were cut from the woven fabric to 100 mm × 100 mm size and stacked into four layers over each other, all oriented at a 0° direction. The influences of pressing temperature, holding time, venting phase during the pressing process and the drying of the textile semi-finished product before the pressing process were investigated. The melting temperature of PLA is 160 °C and degradation of certain components of natural fibres starts around 200 °C. Therefore, only temperatures below 200 °C were chosen.

The parameters for the investigations based on the preliminary tests are given in Table 4. These pressing parameters are specific to the combination of components in the hybrid yarn and the quasi-UD woven fabric, as well as to the stacking characteristics in this study.

**Figure 3:**
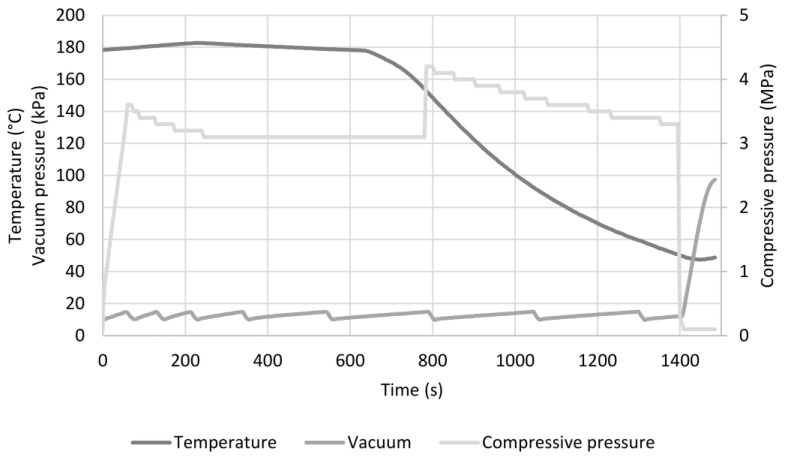
Hot pressing cycle of CO-PLA composite plate.

**Figure 4:**
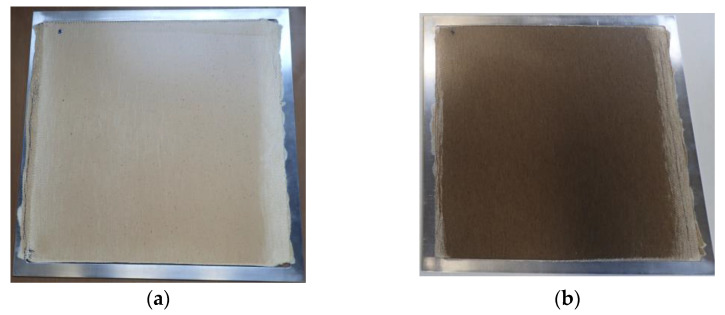
(**a**) Consolidated CO-PLA composite plate with aluminium press frame; (**b**) consolidated FL-PLA composite with aluminium press frame.

Figure 3 shows an example of the pressing cycle of the CO-PLA composite. Temperature and vacuum pressure of the vacuum hot press are plotted on the left axis and compression pressure on the right axis. The two bio-based composites that were produced can be seen in Figure 4a,b.

### 2.5. Charactersiation

#### 2.5.1. Fibre Length Distribution of Hybrid Yarn

The fibre length distribution was determined corresponding to standards DIN 53803 and DIN 53808-1. The length measurement was analysed on the basis of the standards DIN 53804-1 and DIN 53805.

To measure the individual fibre lengths, natural fibres (NF) were extracted from hybrid yarn and laid out on a black velvet covered by a glass plate under a magnifying glass with illumination. With the aid of a tweezer, the NF were separated as carefully as possible and stretched with a second tweezer to determine the true length of the fibre. Every 10th stretched fibre was measured with a ruler. A total of 300 individual fibres were tested.

#### 2.5.2. Tensile Properties of Hybrid Yarn

Tensile tests of the manufactured hybrid yarns were carried out according to DIN ISO 2062 standard using a tensile strength testing device USTER^®^ TENSORAPID 3 from Uster Technologies AG, Uster, Switzerland. Samples of 500 mm length yarn were used. The test velocity was set to 500 mm/min, and the initial load was kept at 0.5 cN/tex. The tensile force versus deformation was recorded and 20 measurements were taken to get the aver-age value for each type of hybrid yarn.

All tests were carried out on conditioned specimens (24 h) at a standard climate of the temperate zone 20 ± 2 °C and humidity 65 ± 2% according to DIN EN ISO 139.

#### 2.5.3. Fibre Mass Fraction and Fibre Volume Fraction

The standard DIN EN ISO 1833-10 describes the extraction of fibres from blends with polylactide from textiles and textile products using dichloromethane triacetate. To deter-mine the mixing ratio, 100 mL of 80% dichloromethane triacetate was added to the sample contained in the Erlenmeyer flask per gram of sample. The flask was shaken manually for 10 min to completely saturate the sample and allowed to stand for 15 min. It was shaken again and again at intervals until all the polylactide had dissolved out of the sample. The contents of the flask were then filtered, and any fibres retained were washed, dried and weighed and thereby the mixing ratio by mass between polylactide and the natural fibre was determined. For each material variant, two samples (double determination) were chosen for the procedure.

Based on the mass fractions and the known densities, the volume fraction of the natural fibre was calculated in the hybrid yarns, woven fabrics and the bio-based composites.

#### 2.5.4. Analysis of Mechanical Properties of Bio-Based Composite

Tensile tests were carried out according to DIN EN ISO 527-5 using a tensile strength testing device, Z100 from ZwickRoell GmbH & Co. KG, Ulm, Germany, with special wedge grips and an external optical strain measuring device. The dimensions of samples were 250 mm × 25 ± 0.2 mm × 2.3 ± 0.2 mm. Test speed was set to 1 mm/min, and the pre-load was kept at 0.1 MPa. The tensile force versus deformation was recorded and 6 measurements were taken to get the average value for each type of bio-based composite. The stress–strain behaviour was evaluated using testXpert^®^II software version 3.71.

The flexural properties of the manufactured composites were determined in a 4-point bending test (method B) on a Zwick Z020 from ZwickRoell GmbH & Co. KG, Ulm, Germany, in accordance with DIN EN ISO 14125 norm. Specimen measurement and automatic calculation of the cross-sectional area-specific flexural modulus took place in conjunction with the testXpert^®^II software. Samples of 60 ± 0.2 mm × 15 ± 0.2 mm × 2.3 ± 0.2 mm were used. Radius of loading and support member were 2 ± 0.2 mm. The test speed was set to 2 mm/min and the pre-load was kept at 0.2 MPa. A total of 6 samples for each material variant were tested.

All tests were carried out in a temperature and relative humidity-controlled laboratory maintained at 20 ± 2 °C and 65 ± 2%, respectively, and on conditioned specimens (24 h) at a standard climate of the temperate zone 20 ± 2 °C and humidity 65 ± 2% according to DIN EN ISO 139 norm.

## 3. Results and Discussion

In the first part of the study, hybrid yarns made from cotton fibre or flax fibre textile waste were mixed with bio-based, thermoplastic matrix fibres and hybrid yarns were produced that were later processed into quasi-UD woven fabrics. The influences of the natural fibres, the yarn structure and the fabric structure on the tensile and flexural properties of the unidirectional composites are analysed in this section.

### 3.1. Yarn Properties

**Figure 5:**
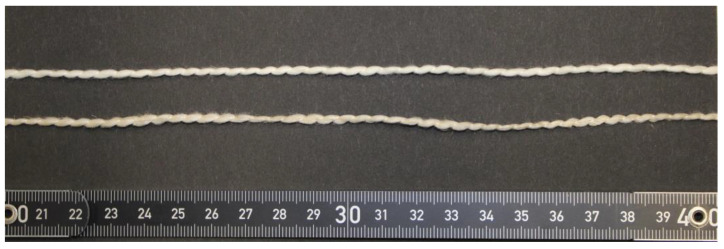
Waste cotton hybrid yarn (above) and waste flax hybrid yarn (below).

The measured textile properties of cotton fibre waste with PLA (CO-PLA), and flax fibre waste with PLA (FL-PLA) are given in Table 5 and Table 6. The visual aspects are shown in Figure 5.

**Figure 6:**
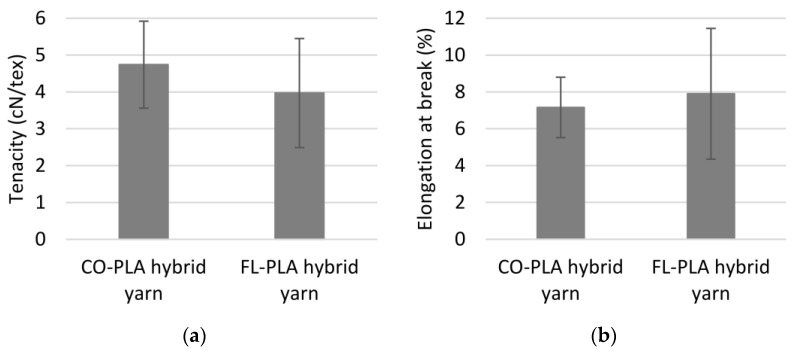
(**a**) Tenacity of hybrid yarns; (**b**) elongation at break of hybrid yarns.

The yarn count of the hybrid yarns is almost the same, 809 tex for the cotton version and 805 tex for the flax version, and the fibre mass content is also similar, 46.57% and 46.36%, respectively. Due to the wrapping process, both hybrid yarns have a similar compact structure in which the inner structure of the blended and oriented core material is preserved, and a comparable hairiness. In detail, the yarn variant with flax shows a significantly higher non-uniformity in mass distribution and long-wave variations between thick and thin sections. The reasons for these are the comparatively stiffer flax fibres and a more inhomogeneous distribution of the flax fibres over the cross-section and length of the yarn. This is also reflected in the tensile properties of the hybrid yarns, as shown in Figure 6a,b. Compared to the cotton hybrid yarn (4.74 cN/tex, 7.16%), the flax hybrid yarn with 3.97 cN/tex has a lower specific tenacity, a higher elongation (7.90%) and significantly higher standard deviations. In addition, due to the fibre mass variations, there is a more uneven distribution of the twists of the wrapping filament over the length of the yarn and, thus, a more irregular compaction of the fibres in the core along the length of the yarn. A reduced frictional pressure between the individual fibres in the yarn and a lower specific strength are the results. The characteristic values for the flax hybrid yarn show a complete overlapping of the fibre properties by the yarn properties. Despite the greater length of the waste flax fibres and the higher fibre strength, the cotton hybrid yarn has the better tensile properties.

**Figure 7:**
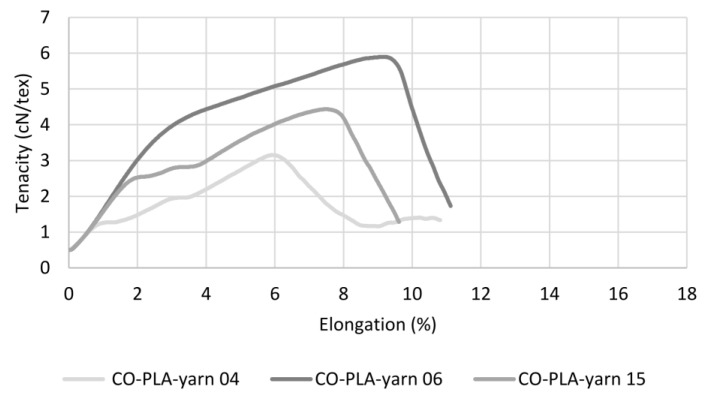
Three selected tenacity–elongation curves of cotton hybrid yarn.

**Figure 8:**
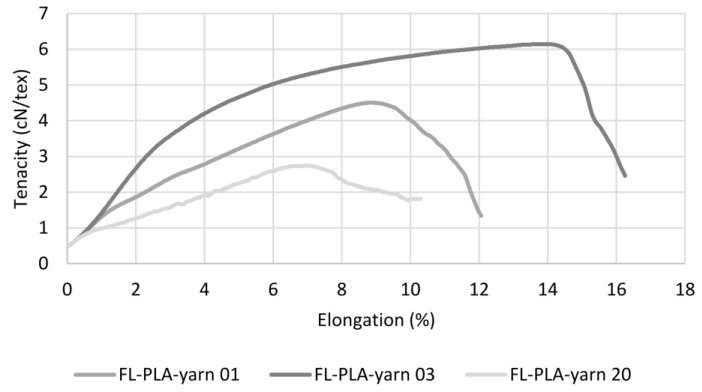
Three selected tenacity–elongation curves of flax hybrid yarn.

Figure 7 shows three examples of the tenacity–elongation curves of the cotton hybrid yarns. The three most important processes in the yarn during the tensile test can be clearly seen from the curves of the cotton hybrid yarns. During the first relatively steep curve, the yarn core is compressed by the wrapping filament, which increases the frictional pressure within the yarn core and between the individual fibres and, thus, aligns the fibres in the direction of the applied force. In the subsequent section, with a lower, partly step-like in-crease, the crimped cotton fibres are stretched, and at the same time, first fibre bundles begin to slip off each other. This sliding is initially hindered by the frictional pressure generated by the wrapping filament, and thus the repeated stick-slip effect occurs. As the wrapping filament stretches in the direction of tension, the frictional pressure on the yarn core increases, resulting in a continued reduction in the yarn diameter. In the last section of the curve, the sliding of the fibre bundles causes more thin spots in the core, while at the same time, the wrapping filament is stretched further and thus blocks the sliding movements of the fibre bundles even more, which causes further thin sections. As the yarn cross-section decreases, less and less force can be transferred, resulting in complete failure of the yarn structure at one of the weak points. In the case of flax hybrid yarns, on the other hand, these mechanisms can only be seen to some extent in the exemplary curves shown in Figure 8. The reason for this is the higher non-uniformity of the mass distribution compared to the cotton hybrid yarn, and, thus, the higher number of thick and thin sections as well as the lack of crimp of the flax fibres. This becomes particularly clear in the two curves at the bottom. There is a direct transition from the phase of fibre alignment to the flax fibre bundles sliding against each other, with corresponding stick-slip effect. This is caused by the frictional pressure of the wrapping filament, the associated reduction in the yarn cross-section, and the low frictional forces between the uncrimped flax fibres, which results in a more uneven and almost linear curve until yarn failure.

From these results, it can be concluded that hybrid yarns based on cotton or flax fibres obtained from textile waste and PLA achieve sufficient strength and flexibility for weaving.

### 3.2. Fabric Properties

The production of the warp was non-problematic with good results. The properties of the woven fabric are given in Table 7. The values of the grammage in Table 7 are calculated from 400 mm × 400 mm pieces. Different weft yarn densities were tried during the weaving process. Weft densities with nine threads per cm or higher resulted in pre-fabric formation during the weaving process, which made it impossible to continue the weaving process after a short time. A weft density of eight weft threads per cm offered a high process stability and gave good results. The weft insertion speed at three picks per minute was relatively low because the weft cutter was not able to cut the fibres correctly at higher machine speeds.

### 3.3. Fibre Volume Fraction

**Figure 9:**
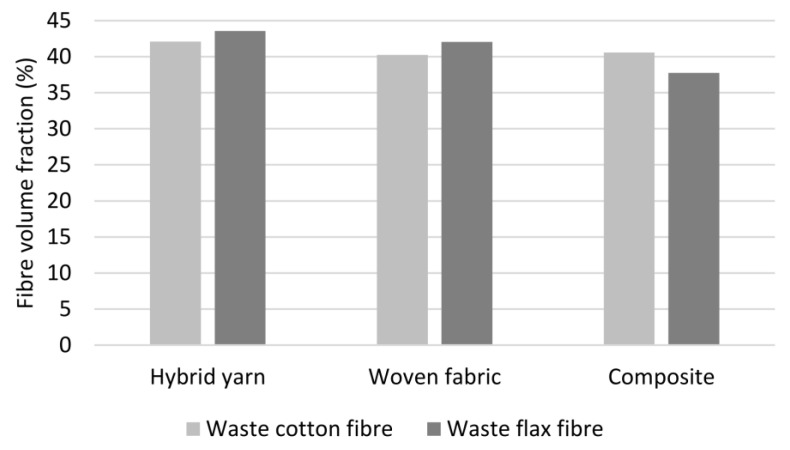
Fibre volume fraction of the semi-finished products from the respective process steps.

Figure 9 shows the determined fibre volume fractions (FVF) for the individual process stages. It can be seen that for the cotton hybrid yarn, the FVF decreases from 42.07 vol.% in the hybrid yarn to 40.25 vol.% in the woven fabric. A reduction in the FVF to a similar extent (from 43.56 vol.% to 42.07 vol.%) can also be observed for the flax variant. This is mainly to the addition of around 1.5 vol.% PLA filament yarn as warp material. A very small amount of natural fibres could also be lost through the abrasion of the very short fibres. The friction between the hybrid yarn weft and the PLA warp yarns during the weft insertion and the beat-up processes results in fibre grinding.

During the consolidation process, there were no physical fibre losses observed with both variants. Tests revealed the FVF of CO-PLA composite remained consistent at 40.58 vol.%. However, the FVF of FL-PLA composite reduced from that of the woven FL-PLA-fabric. Therefore, it can be assumed that the decrease in FVF of the FL-PLA composite is possibly due to the test procedure. Both material variants were tested using the same standard—DIN EN ISO 1833-10—which is valid for both cellulosic and bast fibres. However, in case of FL-PLA composite, the result suggests that in addition to PLA, components of the flax fibres were also decomposed in dichloromethane triacetate. It is conceivable that due to the additional time required to dissolve the consolidated composite in comparison to the non-consolidated hybrid yarn or woven fabric, additional non-cellulosic components such as hemicelluloses, lignin, pectin and/or waxes, which are not contained in cotton fibres or present only in a significantly small proportion, were dissolved out of the bast fibres [28].

### 3.4. Tensile Properties of Composites

**Figure 10:**
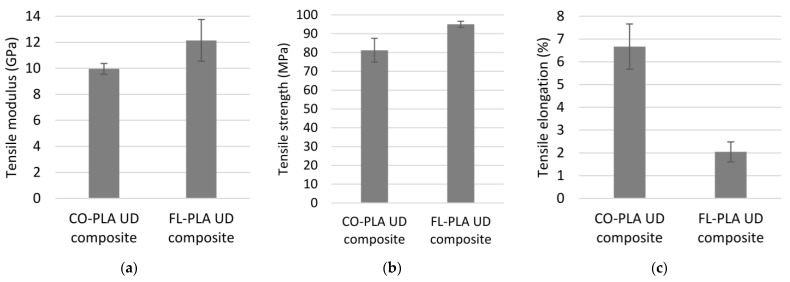
(**a**) Tensile modulus of bio-based composites; (**b**) tensile strength of bio-based composites; (**c**) elongation of bio-based composites.

The tensile modulus and the tensile strength of the composites are shown in Figure 10a,b. The tensile modulus of the cotton variant is 9.95 GPa, which is slightly lower than that of the flax variant (12.14 GPa). Considering the significantly higher standard variation of the FL-PLA composite, both material variants show a similar level of tensile modulus. In terms of tensile strength, the difference between the CO-PLA and FL PLA composites is more evident with 81.22 MPa compared to 95.0 MPa. The difference between the two material variants in both characteristic properties is probably due to the origin of the fibres. Both are recycled fibres obtained from textile waste, which has an effect on the individual fibre lengths, particularly for flax fibres. Waste flax fibres are significantly shorter than technical flax fibres. They are treated several times chemically as well as mechanically in order to be able to be processed in the classical textile processes, which are adapted to the single fibre length of cotton fibres [28].

**Figure 11:**
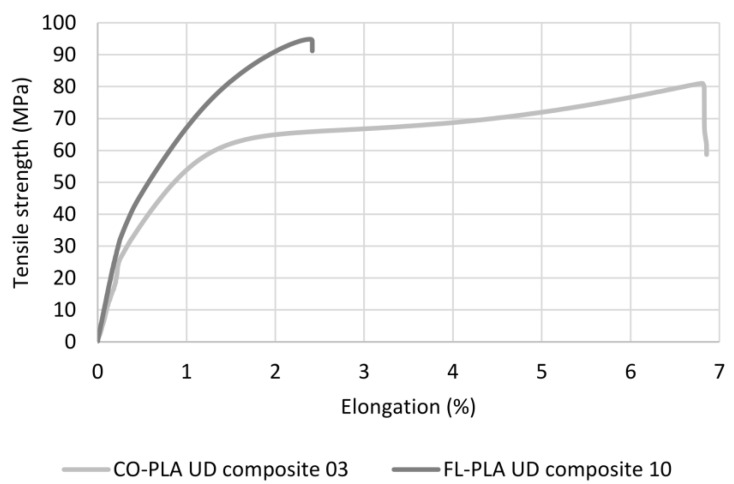
Tensile strength–elongation curve of two selected bio-based composites.

In contrast to the yarn properties, which, at least for the flax hybrid yarn, were deter-mined solely by the yarn structure, the results of the composite properties reflect the in-fluence of the properties of the fibres. The main reason for this is probably the consolidation process, which has largely changed the structure of the hybrid yarns due to the melting of the thermoplastic component and the resulting melt flow process. This is particularly evident in the elongation at break (Figure 10c). The literature indicates an elongation at break in the dry state from 7% to 8% for cotton fibres and 1.2% to 3% for flax fibres [29]. The CO-PLA and FL-PLA composites have elongations at break of 6.67% and 2.04%, respectively, which are within this range. An influence of the elongation at break of the PLA, which is 25 ± 5%, is not noticeable. Flax fibres have a higher tensile strength with lower elongation at break than cotton fibres [29]. On the exemplary stress–strain curve shown in Figure 11, for one sample each of the CO-PLA and the FL-PLA composite, this behaviour can be clearly seen despite the difference in the FVF of around 3 vol.%.

### 3.5. Flexural Properties of Composites

**Figure 12:**
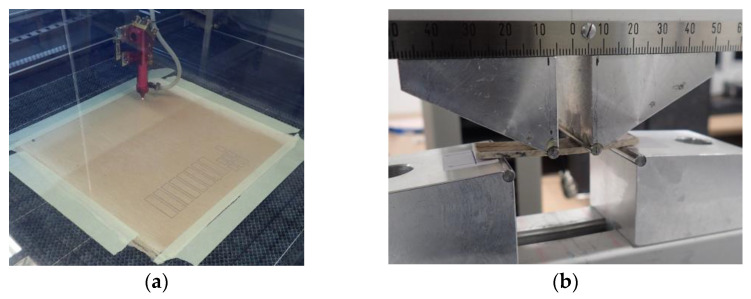
(**a**) Cutting of samples out of CO-PLA composite for four-point bending test; (**b**) four-point bending test of a CO-PLA composite sample.

**Figure 13:**
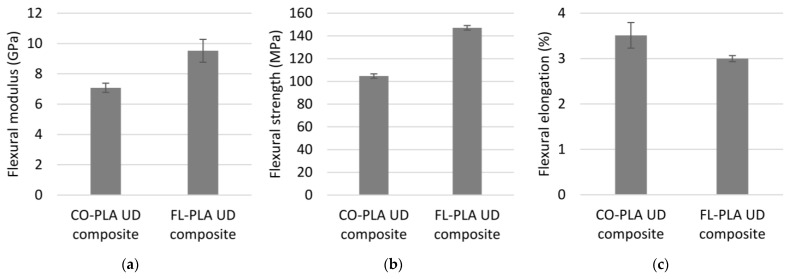
(**a**) Flexural modulus of bio-based composites; (**b**) flexural strength of bio-based composites; (**c**) elongation of bio-based composites.

Figure 12a,b show cutting of the CO-PLA four-point bending test specimens and the test on a CO-PLA sample, respectively. The results of the four-point bending tests for both material variants are given in Figure 13a–c. Unlike the characteristic properties from the tensile tests, the flexural modulus of elasticity and the flexural strength of the FL-PLA composite are better compared to the CO-PLA composites (Figure 13a,b). While the flexural modulus of elasticity for the cotton version is 6.79 GPa, the value for the flax version is 9.87 GPa. The flexural strength of the flax composite is 147.13 MPa, about 29% higher than that of the cotton composite (104.7 MPa). Here, too, the influence of the fibre properties can be seen rather than that of the yarn properties or the yarn structure. Additionally, in contrast to the tensile property, the flexural elongations of both the variants are comparable when the standard deviation is considered (Figure 13c). The CO-PLA composite has a flexural elongation of 3.27% and the FL-PLA composite of 3.07%.

**Figure 14:**
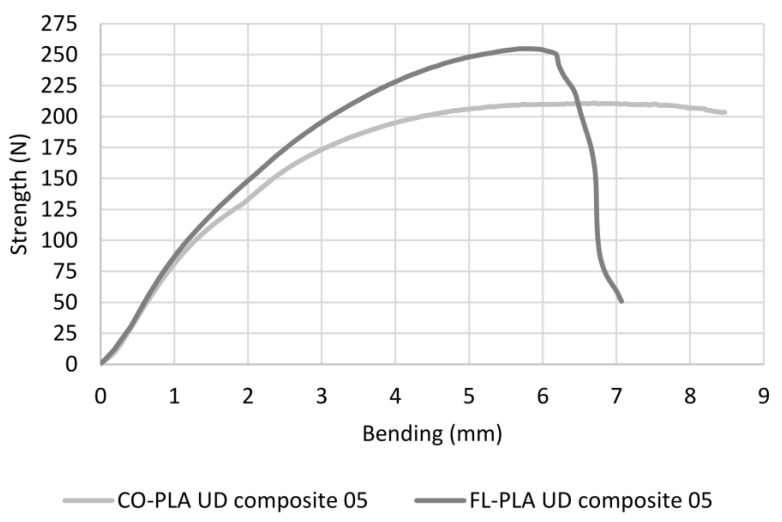
Strength–bending curve of two selected bio-based composites.

Figure 14 shows the strength to bending curve from the four-point bending test for one sample for each material variant. Initially, both show the same increase. While this rise increasingly flattens out in the case of the cotton composite until the end of the test, at a bending of 8.5 mm, there is no clear drop in load and thus no clear sign of breakage. The curve of the FL-PLA composite continues to rise sharply up to a bending of approx. 5.8 mm and breaks. This behaviour of the cotton version is due to the combination of cotton fibres and PLA. In contrast to flax fibres, both cotton fibres and PLA have a comparatively high elongation, so that the composite has a rather ductile fracture behaviour, and the material is torn apart during testing rather than broken. The situation is different with the FL-PLA composite. Here, the PLA content also influences the deflection in the direction of higher elasticity, but this is in turn limited by the low elongation capacity of the flax fibres, which results in a significant load drop at break.

### 3.6. Comparison of Mechanical Properties

A comparison with other composite materials, some of them green, investigated in the literature shows that the tensile and bending properties of the CO-PLA composites and the FL-PLA composites are above those of fibre mats, and they are in the range of the characteristic values of other already optimised hybrid yarns such as commingled or friction yarns. A selection is given in Table 8.

## 4. Conclusions

This research paper reports on the successful manufacturing of hybrid yarns, woven fabrics and composites from recycled natural fibres from textile waste and bio-based thermoplastic fibres. Understanding the different manufacturing stages and the influence of process parameters on the properties of yarns, woven fabrics and composites enables the production of materials that better meet the requirements of the final application.

Hybrid yarns were produced on a hollow spindle spinning machine by wrapping a thermoplastic PLA multifilament around a fine fibre tow exiting the drafting device. This fine fibre tow consisted of a mixture of cotton or flax fibres obtained from textile waste and PLA staple fibres. The hybrid yarns exhibited sufficient elongation behaviour and good strength with a fibre volume content of more than 40 vol.%, which made weaving possible.

Subsequently, quasi-unidirectional woven fabrics were produced from the hybrid yarns. The weft densities of the fabrics are eight yarns per cm and the warp densities of the fabrics are 11 yarns per cm. The hybrid yarns were used as weft material, while the PLA filament served as the warp. The weft density of eight yarns per cm is the maximum attainable weft density for both fibres. The width of the woven fabrics was 50 cm.

The quasi-UD woven fabrics were consolidated into composites in a hot pressing process and analysed with regard to their tensile and flexural properties. An influence of the yarn structure or the yarn properties could not be determined. Rather, the properties of the fibres could be clearly seen in the composite characteristics. In addition, the results for the flax composite showed an influence of the origin of the flax fibre from a textile application.

Thus, this study has clearly shown that the production of semi-finished products from textile waste for applications in the fields of lightweight construction and fibre composite plastics is a potential process for the upcycling of waste materials.

Future works aim to investigate further yarn, fabric and composite parameters in or-der to further adapt bio-based semi-finished products from recycled waste to meet the given requirements and optimise them with a view to use them in lightweight construction applications. Based on the mechanical properties achieved in this first study, the material can be used as a substitute for other natural fibre materials. Other fields of application could be, for example, agriculture and forestry, where the biodegradability, which still has to be investigated, could be an advantage. In addition, the thermomechanical behaviour of hybrid yarns and blended fabrics will be tested at different temperatures, including the melting temperature of the bio-polymer used, to gain a better understanding of the behaviour of these materials at high temperatures.

## Figures and Tables

**Table 1:** Materials used for manufacture of hybrid yarns.

Material	Supplier/Origin	Fineness	Density	Melting Point
Waste cotton fibre	Spinning production waste		1.5 g/cm³	
Waste flax fibre	Spinning production waste		1.4 g/cm³	
PLA staple fibres	TREVIRA^®^ GmbH	1.7 dtex	1.25 g/cm³	160 °C
PLA filament yarn (Fully orientated yarn)	TREVIRA^®^ GmbH	167 dtex f48	1.25 g/cm³	160 °C

**Table 2:** Hybrid yarn properties.

Spinning/Yarn Parameter	Value
Natural fibre	Waste cotton fibre (CO) or waste flax fibre (FL)
Bio-based thermoplastic staple fibre	PLA
Bio-based thermoplastic wrapping filament	PLA
Fibre mass fraction (target)	50 wt.%
Card sliver	5.5 ktex
Draw frame sliver	5 ktex
Yarn count (target)	800 tex
Wrap count	150 T/m
Break draft	1.5 fold
Main draft	4.2 fold
Field width	50 mm
Production speed	12 m/min

**Table 3:** Weaving parameters of quasi-UD woven fabrics.

Weaving Parameters	Quasi-UD CO-PLA	Quasi-UD FL-PLA
Warp material	PLA filament yarn	PLA filament yarn
Warp density	11 yarns/cm	11 yarns/cm
Warp tension	5 cN	5 cN
Weft material	CO-PLA hybrid yarn	FL-PLA hybrid yarn
Weft density	8 yarns/cm	8 yarns/cm
Weft insertion speed	3 picks/min	3 picks/min
Width	0.50 m	0.50 m

**Table 4:** Pressing parameters.

Pressing Parameter	Preliminary Tests	This Study
Temperature	180–190 °C	180 °C
Holding time	10–20 min	10 min
Vacuum	100 mbar	100 mbar
Compressive pressure	3.1 MPa	3.1 MPa
Predrying temperature	None/80 °C	80 °C
Predrying time	None/120 min	120 min
Venting phase	None/Yes	None

**Table 5:** Statistics of individual fibre length of waste cotton fibres and waste flax fibres in hybrid yarn.

Fibre Parameter	Waste Cotton Fibre	Waste Flax Fibre
Arithmetical mean (mm)	22.87	29.62
Standard deviation (mm)	10.23	13.49
Variance (%)	44.75	45.53
Median (mm)	24.00	37.00
Minimum (mm)	2.00	4.00
Maximum (mm)	42.00	56.00
Short fibre content ≤12 mm (%)	18.30	17.00

**Table 6:** Textile properties of hybrid yarns.

Hybrid Yarn	Linear Density (Tex)	Natural Fibre Mass Fraction (%)	Variation Coefficient of Mass (%)
CO-PLA	809 ± 5	46.57	6.81
FL-PLA	805 ± 5	46.36	11.15

**Table 7:** Textile properties of woven fabrics.

Woven Fabric	Grammage (g/m²)	Fibre Mass Fraction (%)	Fibre Volume Fraction (%)
CO-PLA twill 2/2	712.65	44.70	40.25
FL-PLA twill 2/2	725.36	44.85	42.07

**Table 8:** Mechanical properties of selected natural fibre reinforced thermoplastic composites.

Reinforcement	Matrix	Reinforcement Type	Tensile Modulus (GPa)	Tensile Strength (MPa)	Flexural Modulus (GPa)	Flexural Strength (MPa)	References
Waste cotton fibre	PLA	Filament wrapping yarn	9.3–10.6	69–89	6.6–7.5	102–107	This study
Waste flax fibre	PLA	Filament wrapping yarn	8.9–13.2	93–98	8.3–10.4	143–149	This study
Recycled flax fibre	Poly propylene (PP)	UD fabric	1.6	26			[30]
Recycled flax fibre	Polyamide 11	UD fabric	1.1	45			[30]
Flax	PP	Friction spun hybrid yarn	8–15	80–128	9–17	95–145	[31]
Flax	PP	Non-woven commingled fabric	1.8–8.7	12–143			[32,33]
Jute	PP	Commingled yarn	2.5–3.3	53–68	2.7–4.5	53–75	[34]
Flax	PP	Woven commingled fabric	2.3–3.8	20–30			[35]
Flax	PLA	Woven commingled fabric	3.1–3.5	8–11			[35]
Jute	PLA	Short fibres	5.2–8	151–205	10.5–15	180–225	[36]
Flax	PP	Yarns/interwoven fabric	0.9–5.8	7–71	0.6–8.4	16–40	[37]
Flax	PP	Non-woven fabric	1.2–1.7	32–80	1.5–6.2	30–75	[38]
Flax	PP	Commingled yarn/woven fabric	4.3–10.7	46–92			[39]
Flax	PP	Non-woven commingled fabric	1.2–5.5	27–62	1.9–9.3	20–72	[40]

## Data Availability

Data are contained within this article.
